# The role and significance of the PI3K/Akt-mTOR signaling pathway in infectious diseases

**DOI:** 10.3389/fcimb.2026.1782712

**Published:** 2026-03-30

**Authors:** Ganggang Sheng, Chaopeng Li, Da Liu, Weishan Wang, Nannan Pang, Zhendong Zhang

**Affiliations:** Department of Orthopedic Surgery, The First Affiliated Hospital of Shihezi University, Shihezi, China

**Keywords:** immune regulation, infectious diseases, pathogen evasion, PI3K/Akt-mTOR, targeted therapy

## Abstract

The PI3K/Akt-mTOR signaling pathway, as a highly conserved and crucial intracellular signal transduction network, is deeply involved in core biological processes such as cell metabolism, proliferation, survival, and immune responses, playing a pivotal role in the occurrence and development of infectious diseases. This review comprehensively and systematically elaborates on the molecular composition, activation mechanisms, and negative regulatory mechanisms of the pathway, with a particular focus on analyzing its key regulatory role in the anti-infective responses of host immune cells (macrophages, T cells, NK cells, dendritic cells, B cells, etc.), as well as the mechanisms by which pathogens (bacteria, viruses, parasites, fungi, etc.) hijack and interfere with this pathway. Research has shown that the dynamic balance of the PI3K/Akt-mTOR pathway is one of the key factors determining the outcome of infection: the host enhances immune defense by activating this pathway, while pathogens achieve immune evasion by targeting key molecules in the pathway. Furthermore, this review systematically integrates the dual role of the PI3K/Akt-mTOR pathway in infection immunity, aiming to elucidate its central position as a core for host defense and pathogen evasion, and to provide a clear theoretical framework for subsequent targeted therapeutic research.

## 1 Introduction

Infectious diseases, caused by the invasion of pathogens such as bacteria, viruses, and parasites into the host, are fundamentally characterized by a continuous and complex dynamic game between the host immune response and pathogen evasion. In this process, the precise regulation of intracellular signaling pathways plays a decisive role. The PI3K/Akt-mTOR signaling pathway is a core hub for cells to sense external environmental signals (such as nutrients, cytokines, and pathogen stimulation). Through orderly cascade reactions, it comprehensively regulates key processes like cellular metabolic reprogramming, immune cell activation, and effector function execution, thereby directly influencing the outcome of infection (Zhang et al., 2019; Liu and Sabatini, 2020; Tewari et al., 2022). Thus, the PI3K/Akt-mTOR signaling pathway, as a core intracellular signal hub, plays a key role in transducing pathogen-derived stimulation signals to initiate and shape host immune responses, and its dysregulation is closely related to the occurrence and development of infections.

In recent years, numerous studies have gradually revealed the effects of the PI3K/Akt-mTOR pathway in infectious diseases: on one hand, the host can activate this pathway to effectively promote the proliferation and differentiation of immune cells, significantly enhancing the ability to clear pathogens; on the other hand, various pathogens have evolved over time to develop strategies targeting this pathway, successfully achieving self-replication by inhibiting immune responses or hijacking cellular resources (Donia et al., 2010; Mammana et al., 2018; Zhang et al., 2019; Sabbah et al., 2021; Ma et al., 2023). For example, Mycobacterium tuberculosis inhibits Akt phosphorylation in macrophages by secreting effector proteins, thereby weakening their bactericidal function. It is worth noting that the degree of inhibition of Akt activity may vary among different Mycobacterium tuberculosis strains (e.g., clinical isolates vs. laboratory strains), and this inhibitory effect may also change dynamically at different stages of infection (early vs. chronic phase), reflecting the adaptability and context-dependence of pathogen strategies (Brufsky and Dickler, 2018); whereas rotavirus is highly dependent on this pathway to maintain its own replication cycle (Yin et al., 2018). A deep understanding of the mechanism of the PI3K/Akt-mTOR pathway in infection is of great significance for developing new therapies with both immunomodulatory and anti-infective functions, providing key targets and new ideas.

This review will adopt the perspective of “host-pathogen interplay” to systematically elaborate: (1) How the host utilizes this pathway to construct immune defense; (2) How different pathogens have evolved diverse strategies to attack this pathway; (3) Based on the above interplay mechanisms, how to design precise intervention strategies. By exploring these three progressive questions, we aim to reveal the central role of this pathway in infection and its prospects for clinical translation.

## 2 Core components and regulatory mechanisms of the PI3K/Akt-mTOR signaling pathway

### 2.1 Molecular composition of the pathway

The PI3K/Akt-mTOR pathway is composed of three core molecules: phosphatidylinositol 3-kinase (PI3K), protein kinase B (Akt, also known as PKB), and mammalian target of rapamycin (mTOR) (Fattahi et al., 2020). Having clarified the key roles of PI3K, Akt, and mTOR in signal transduction, and how they regulate important biological processes such as cell survival, metabolism, and proliferation through complex interactions (Yu et al., 2022), we further explore the specific role of this pathway in cellular function. PI3Ks are a class of lipid kinases that can be further divided into classes I, II, and III based on structural and functional differences (Engelman et al., 2006; Bilanges et al., 2019). Among them, class I PI3Ks (e.g., PI3Kδ, PI3Kγ) are highly expressed in immune cells and can be activated by tightly binding to activated receptor tyrosine kinases or G protein-coupled receptors, subsequently catalyzing the conversion of phosphatidylinositol-4,5-bisphosphate (PIP2) into the second messenger phosphatidylinositol-3,4,5-trisphosphate (PIP3) (Fresno Vara et al., 2004; Carnero et al., 2008; Shen et al., 2022; Wang et al., 2024).

Akt, a key downstream target of PI3K, specifically binds to PIP3 via its PH domain and localizes precisely to the cell membrane (Revathidevi and Munirajan, 2019). Subsequently, through the synergistic action of phosphoinositide-dependent kinase 1 (PDK1) and mTOR complex 2 (mTORC2) (Sun et al., 2020), Akt is phosphorylated at Thr308 and Ser473 residues, respectively, thereby becoming activated (Janecka-Widła et al., 2022). Activated Akt then extensively and deeply regulates a series of important biological processes, including cell survival, metabolism, and proliferation, by phosphorylating numerous downstream target proteins (Buchkovich et al., 2008; Ferrara et al., 2023).

mTOR is a serine/threonine kinase that exerts its biological functions by forming two functionally distinct complexes: mTORC1 and mTORC2 (Jhanwar-Uniyal et al., 2019; Szwed et al., 2021). mTORC1, composed of mTOR, Raptor, mLST8, and other key molecules, is highly sensitive to nutrient signals and primarily regulates protein synthesis and autophagy by phosphorylating p70S6K and 4E-BP1 (Fu and Hall, 2020; Chen et al., 2022). mTORC2, containing mTOR, Rictor, mSin1, and other molecules, is mainly involved in cytoskeletal remodeling and the phosphorylation activation of Akt, playing an indispensable role in maintaining cell morphology and functional stability (Martelli et al., 2018).

### 2.2 Regulatory network of the pathway

The activity of the PI3K/Akt-mTOR pathway is strictly controlled by extremely precise and complex regulatory mechanisms within the cell. The phosphatase PTEN, as the main negative regulator of the pathway, efficiently dephosphorylates PIP3 back to PIP2, thereby directly antagonizing the action of PI3K and precisely regulating the intensity of pathway activity. Additionally, the tuberous sclerosis complex (TSC1/TSC2) negatively regulates mTORC1 by inhibiting the activity of the small G protein Rheb (Inoki et al., 2003; Tee et al., 2022; Eser et al., 2024). Under conditions of cellular energy stress, AMPK can rapidly phosphorylate TSC2, significantly enhancing its inhibitory function, ensuring that the cell adjusts the activity of the PI3K/Akt-mTOR pathway in a timely manner under unfavorable conditions like energy deficiency, thus maintaining intracellular homeostasis (Wei et al., 2023; Wang et al., 2024). Negative regulatory mechanisms represented by PTEN, the TSC complex, and AMPK collectively constitute a safety valve that maintains the dynamic balance of this pathway and prevents overactivation.

Furthermore, to deeply understand the functional complexity of this pathway, one must distinguish the different functional modules of its core components. mTORC1 and mTORC2 play distinct but sometimes interrelated roles in immune regulation: mTORC1, as a sensor of nutrients and energy, dominates protein synthesis, autophagy inhibition, and the differentiation of effector T cells like Th1 and Th17; whereas mTORC2, primarily through phosphorylation of Akt at Ser473, influences cytoskeletal remodeling, metabolic stability, and the functional maintenance of regulatory T cells (Tregs). Within the same immune process, they may be differentially regulated and exert synergistic or antagonistic effects. For example, in early T cell activation, mTORC2 assists in the complete activation of Akt, while mTORC1 drives subsequent clonal expansion. Similarly, the expression patterns and functions of different PI3K isoforms are highly specific: for instance, PI3Kδ is highly expressed mainly in lymphocytes and is crucial for B cell receptor (BCR) and T cell receptor (TCR) signaling; while PI3Kγ is enriched in myeloid cells (e.g., neutrophils, macrophages) and is a core molecule downstream of chemokine receptors (GPCRs) regulating cell migration. This difference in subcellular localization, expression profile, and signal coupling determines their non-interchangeable functions in infection immunity.

**Figure 1:**
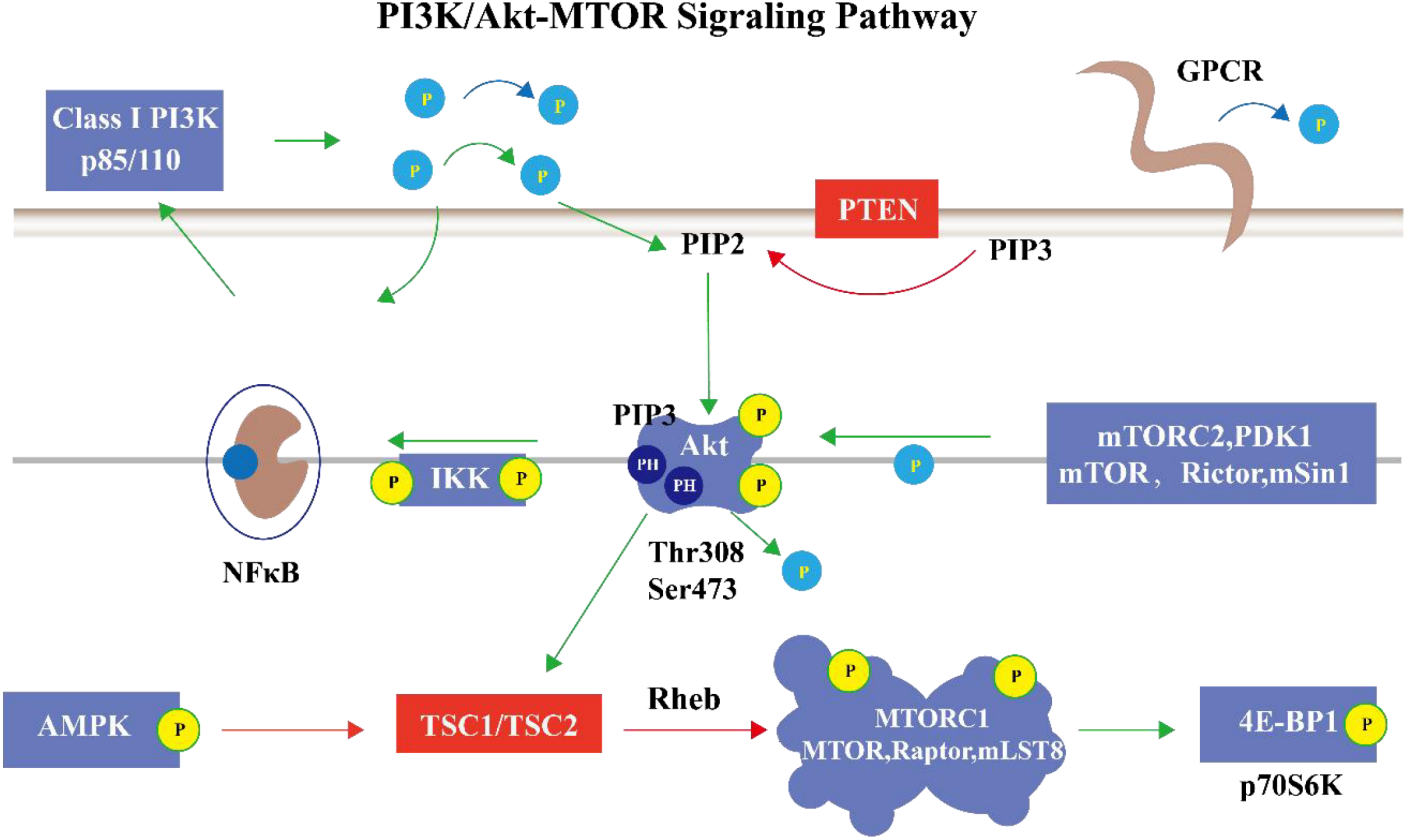
Molecular composition and signal flow of the PI3K/Akt-mTOR signaling pathway. The pathway originates at receptor tyrosine kinases or G protein-coupled receptors on the cell membrane. Upon activation, they recruit and phosphorylate the p85/p110 subunits of class I PI3K. Activated PI3K catalyzes the phosphorylation of PIP_2_ to generate PIP_3_. PTEN acts as a negative regulator, dephosphorylating PIP_3_ back to PIP_2_. PIP_3_ recruits Akt and PDK1 to the cell membrane. PDK1 phosphorylates Akt at Thr308, while the mTORC2 complex phosphorylates Akt at Ser473, leading to its full activation. Activated Akt phosphorylates and inhibits the TSC1/TSC2 complex, thereby relieving its GAP activity inhibition on the small G protein Rheb. Rheb-GTP then directly activates the mTORC1 complex. mTORC1 promotes ribosome biogenesis and protein translation initiation by phosphorylating downstream effectors p70S6K and 4E-BP1. Additionally, activated Akt can directly phosphorylate and inhibit pro-apoptotic proteins and activate the NF-κB pathway, promoting the production of pro-inflammatory cytokines, collectively regulating cell survival. AMPK, activated during energy stress, can phosphorylate and enhance TSC2 activity, thereby inhibiting mTORC1, forming an energy-sensing negative feedback loop. The figure illustrates the complete linear signal cascade from receptor activation to functional outputs (cell survival, proliferation, metabolism, and autophagy inhibition) and its key regulatory nodes.

This pathway engages in extensive and deep cross-talk with other signaling networks. For example, the NF-κB pathway can enhance Akt activity by upregulating PI3K expression; conversely, Akt can effectively activate NF-κB by phosphorylating IκB kinase (IKK). Their synergistic action promotes the secretion of pro-inflammatory factors, playing a key regulatory role in immune inflammatory responses (Bhatt and Damania, 2012). In immune cells, TCR/BCR signals can activate the PI3K/Akt-mTOR pathway and cooperate with the MAPK pathway to precisely regulate cell activation and differentiation, ensuring that immune cells mount appropriate and effective responses to pathogen stimulation (Chapman et al., 2015). This extensive cross-talk implies that the PI3K/Akt-mTOR pathway does not operate in isolation but is a highly integrated node within the immune signaling network, and its functional output depends on the synergy and balance of multiple pathways. (**Figure 1**).

### 2.3 Summary of mechanisms and unresolved questions

In summary, the activity of the PI3K/Akt-mTOR pathway is finely regulated by multiple negative feedback mechanisms including PTEN, the TSC complex, and AMPK, and exhibits extensive cross-talk with signaling networks such as NF-κB and MAPK. However, several key questions regarding the regulation of this pathway during infection remain unresolved: (1) How is the activity of PTEN dynamically regulated upon stimulation by different pathogens? Are there pathogen-encoded PTEN mimics or antagonists? (2) The spatiotemporally specific activation patterns of mTORC1 and mTORC2 in immune cells remain unclear – how are they differentially regulated at different stages of infection and in different subcellular localizations of the cell? (3) How is the cross-talk between this pathway and other signaling networks integrated within the infection microenvironment? When multiple signals are activated simultaneously, how does the cell “compute” and output a unified biological decision? Answering these questions will provide a deeper mechanistic basis for understanding the complex functions of this pathway in infection.

## 3 The core role of the PI3K/Akt-mTOR signaling pathway in host immune responses

The PI3K/Akt-mTOR pathway is a central signaling hub coordinating immune cell responses. Its activity dynamically regulates the development, differentiation, metabolism, and effector functions of cells ranging from myeloid lineages (such as macrophages, dendritic cells, neutrophils) to lymphoid lineages (including T cells, B cells, NK cells, various innate lymphoid cells (ILC), and innate-like lymphocytes (ILL) such as NKT cells, MAIT cells, etc.) (Deane and Fruman, 2004; Owen et al., 2019). The following sections will analyze how the host constructs regulatory mechanisms through this pathway from the perspective of both myeloid and lymphoid immune cells.

### 3.1 Regulation of myeloid innate immune cell function

#### 3.1.1 Regulation of macrophage function

Macrophages, as the first line of defense in innate immunity, have their polarization state and function strictly and finely regulated by the PI3K/Akt-mTOR pathway. Upon stimulation with Toll-like receptor agonists like LPS, activation of the PI3K/Akt-mTOR pathway drives macrophages toward a pro-inflammatory M1-like phenotype, promoting the production of pro-inflammatory factors such as TNF-α and IL-6, and enhancing phagocytic and bactericidal functions; whereas IL-4/IL-13 stimulation is often accompanied by inhibition of this pathway, favoring polarization towards an M2-like phenotype involved in tissue repair and immune regulation (Vergadi et al., 2017). However, this relationship is not fixed – the ultimate functional output of this pathway in macrophages is highly dependent on microenvironmental signals, and its sustained activation can, in some contexts, even induce a mixed phenotype with reparative features, suggesting the plasticity and context-dependence of this pathway in determining macrophage functional fate.

In the infection microenvironment, the dynamic balance of the PI3K/Akt-mTOR pathway is decisive for the anti-infection efficiency of macrophages. For example, in *Staphylococcus aureus* infection, activation of PI3K/Akt-mTOR significantly enhances ROS production and bacterial clearance by macrophages, effectively curbing bacterial proliferation and spread (Chen et al., 2022). In contrast, during *Mycobacterium tuberculosis* infection, the pathogen cleverly inhibits Akt phosphorylation by secreting effector proteins, severely impairing macrophage bactericidal function, leading to a persistent infection state, posing a great challenge to treatment (Zhou et al., 2020; Sha et al., 2021; Ferrara et al., 2023). Furthermore, this pathway profoundly influences the efficiency of macrophage clearance of intracellular pathogens by precisely regulating autophagy. Specifically, inhibition of mTORC1 can induce autophagosome formation, promoting the degradation of intracellular pathogens like *Mycobacterium tuberculosis* and *Listeria monocytogenes*, providing an important guarantee for host defense against infection (Li et al., 2015; Narasimhan et al., 2016; Zhou et al., 2018). The activity of this pathway in macrophages is directly linked to their antibacterial capacity, and pathogen interference with the pathway is a significant means of successful immune evasion.

In summary, the function of the PI3K/Akt-mTOR pathway in macrophages is not a simple M1/M2 switch. Its ultimate effect depends on the stimulus (e.g., LPS vs. IL-4), signal strength and duration, and cross-talk with other pathways (e.g., JAK/STAT, NF-κB). For example, moderate mTORC1 activation promotes M1, but excessive or sustained activation may lead to metabolic exhaustion and dysfunction. Future research needs to elucidate the specific contributions of different PI3K isoforms (e.g., PI3Kγ in chemotaxis vs. PI3Kδ in polarization) in this process.

#### 3.1.2 Regulation of dendritic cells

The antigen presentation function of dendritic cells (DCs) is also strictly regulated by this pathway. Activation of mTORC1 promotes DC maturation and the expression of co-stimulatory molecules (CD80, CD86), significantly enhancing T cell activation and effectively initiating adaptive immune responses; whereas inhibition of mTOR renders DCs a tolerogenic phenotype, inducing immune tolerance and preventing excessive immune responses that could damage the body. In acute lung injury, high mobility group box 1 protein (HMGB1) promotes DC maturation by activating the PI3K/Akt-mTOR pathway, exacerbating pulmonary inflammation (Li et al., 2015; Narasimhan et al., 2016; Zhou et al., 2018; Sha et al., 2021), while pathway inhibitors can reduce tissue damage, providing new strategies and targets for the treatment of acute lung injury.

Plasmacytoid dendritic cells (pDCs), a special subset of DCs, are the main producers of type I interferons (IFN-I) and play a crucial role in antiviral immunity. The burst production of IFN-I induced by TLR7/9 signaling is a highly regulated process involving the complex integration of multiple signaling pathways. Research indicates that the PI3K/Akt pathway is involved, but its precise role is far from elucidated and exhibits significant mechanistic intertwining (Swiecki and Colonna, 2015). On one hand, PI3K/Akt may provide a permissive basis for sustained IFN-I production by regulating pDC survival, metabolism, and migration. On the other hand, whether it directly participates in the transcription of Ifn genes or functions through positive or negative feedback loops with the IRF7-dependent pathway (the classical core pathway for IFN-I production in pDCs) remains controversial. More complexly, some observed effects of PI3K/Akt may be achieved through mTOR-independent routes, further blurring its exact position in the regulation of pDC function. Therefore, clarifying whether PI3K/Akt signaling acts as a core driver, a co-regulator, or an indirect supporter in pDCs, as well as its hierarchical relationship and spatiotemporal interaction with other key signaling modules like IRF7 and NF-κB, is an important question for future research.

#### 3.1.3 Neutrophils

Neutrophils are core effector cells in anti-bacterial infection. Studies have shown that the PI3Kγ subunit plays a key role in neutrophil chemotaxis. PI3Kγ activation mediated by G protein-coupled receptors (GPCRs) catalyzes the generation of PIP3, guiding neutrophils to migrate precisely to the site of infection (Hirsch et al., 2000). PI3K signaling is crucial for the chemotactic ability of these rapidly moving cells by regulating the cytoskeleton and polarity.

#### 3.1.4 Other myeloid cells

Mast Cells: Mast cells are central effector cells in allergic reactions and also participate in anti-parasitic immunity and innate immune defense. FcϵRI cross-linking or other activation signals strongly activate the PI3K/Akt pathway, thereby regulating mast cell degranulation, cytokine production, and survival, playing a dual role in defense and pathological processes (Vosseller et al., 1997). This indicates that the PI3K/Akt pathway is a core signal amplifier for mast cells responding to stimuli and releasing effector molecules.

Eosinophils and Basophils: These two cell types play roles in anti-parasitic infections and allergic diseases. The PI3K signaling pathway is involved in mediating eosinophil chemotaxis, degranulation, and survival. For basophils, PI3K/Akt is one of the key signals mediating the rapid secretion of IL-4/IL-13 and pro-inflammatory responses (Sullivan and Locksley, 2009). Therefore, this pathway also regulates the recruitment and function of granulocyte subsets in specific types of infections and inflammatory reactions.

### 3.2 Regulation of lymphoid immune cell function

#### 3.2.1 Adaptive immune lymphocytes

The PI3K/Akt-mTOR pathway is a key regulator in T cell activation and differentiation. Upon specific binding of the T cell receptor (TCR) to the peptide-MHC complex, PI3K is rapidly activated via adaptor proteins, which effectively promotes Akt/mTORC1 signaling, significantly enhancing the expression of glucose transporter 1 (GLUT1) and the level of glycolytic metabolism (Zhang et al., 2021). It captures glucose and converts it to lactate, which can be used for oxidative phosphorylation even in the presence of oxygen (Gerriets et al., 2015). Glucose is first converted to glucose-6-phosphate (G-6-P), then to fructose-6-phosphate (F-6-P), and then to F-1,6-BP via PFK1, a key regulatory enzyme in glycolysis (Adeva-Andany et al., 2016). Next, F-1,6-BP enters the second part of glycolysis, ultimately producing ATP and pyruvate (Icard et al., 2021). Therefore, glycolysis greatly contributes to T cell proliferation and differentiation, providing ample energy and biosynthetic precursors for the rapid proliferation of T cells, meeting their high metabolic demands during immune responses.

Differences in pathway activity can precisely determine the differentiation direction of T cell subtypes: activation of mTORC1 effectively promotes the differentiation of Th1 and Th17 cells, enhancing cellular immune responses to effectively combat intracellular pathogen infections; while inhibition of mTORC1 favors the generation of Treg cells, exerting immunomodulatory effects and preventing excessive immune reactions that could damage the body. In infectious diseases, this regulatory mechanism is crucial for maintaining immune balance. For example, in *Helicobacter pylori* infection, activation of the PI3K/Akt-mTOR pathway can promote IFN-γ secretion by Th1 cells, enhancing bacterial clearance and effectively controlling the infection (Xie et al., 2014). In chronic viral infections, persistent antigen stimulation leads to sustained activation of the PI3K/Akt-mTOR pathway by TCR signals. This, intertwined with the upregulation of inhibitory receptors (like PD-1) and metabolic reprogramming, collectively drives the occurrence of T cell exhaustion. It is currently believed that excessive mTORC1 activity may accelerate the terminal differentiation and functional loss of T cells by promoting glycolysis and inhibiting mitochondrial biogenesis (Xia et al., 2020), leading to a significant decline in the body’s immune defense against the virus, allowing the virus to replicate and spread persistently.

T follicular helper cells (Tfh) are a key subset that assists B cells in maturing within germinal centers and producing high-affinity antibodies. Their differentiation and function are highly dependent on the PI3K-Akt-mTOR signaling network (Faliti et al., 2024). The activation level of Akt finely balances the differentiation fate of Tfh cells versus other CD4+ T cell subsets by regulating transcription factors such as FOXO1, which is crucial for effective humoral immunity (Essig et al., 2017; Künzli et al., 2020). This indicates that the pathway not only regulates effector T cells but is also deeply involved in the specialization of helper T cell subsets, thereby indirectly regulating the quality of humoral immunity.

Tissue-resident memory T cells (TRM) are long-term “sentinels” stationed in barrier tissues (e.g., skin, lung, gut), providing rapid local protection. mTOR signaling has been shown to be necessary for the formation, metabolic adaptation, and maintenance of TRM cells, possibly through sensing local microenvironmental nutrient conditions (Pan et al., 2017). This suggests a unique role for the mTOR pathway in the tissue residency and long-term functional maintenance of memory T cells.

Proliferation, differentiation, and antibody secretion of B cells are also finely regulated by the PI3K/Akt-mTOR pathway. In the extrafollicular B cell response, binding of antigen to the B cell receptor (BCR) rapidly activates the PI3K/Akt-mTOR pathway. The activation strength of this pathway precisely determines whether B cells differentiate into germinal center (GC) B cells (Staniek et al., 2024), producing high-affinity antibodies through somatic hypermutation for long-term immune protection (Staniek and Rizzi, 2025), or follow the extrafollicular (EF) pathway to rapidly form antibody-secreting cells (ASCs), generating short-lived plasma cells for a quick response to infection. When the signal is too strong, B cells tend towards the EF pathway, rapidly producing antibodies to quickly clear pathogens; moderate signaling promotes GC formation (Cyster and Allen, 2019), generating more efficient immune protection through complex immune reactions.

In infectious diseases, this pathway is crucial for B cell-mediated immune protection. For example, in neonatal sepsis, B cell-activating factor (BAFF) promotes the differentiation of peripheral blood B cells into CD19^+^CD24^bigh^CD38^bigh^ regulatory B cells (Bregs) by activating the PI3K/Akt-mTOR pathway (Liao et al., 2023), which in turn suppresses excessive inflammatory responses and maintains immune balance by secreting IL-10. This reveals another dimension of this pathway’s role in B cell function: participation in the differentiation of regulatory B cells to control inflammatory damage during infection.

#### 3.2.2 Innate lymphoid cells

The cytotoxic activity and cytokine secretion function of NK cells are highly dependent on the activation of the PI3K/Akt-mTOR pathway. IL-15, by activating this pathway, effectively promotes metabolic reprogramming in NK cells, significantly enhancing their killing ability against virus-infected cells and tumor cells, playing an important role in the body’s antiviral and antitumor immunity (Zhu et al., 2020). In an infectious osteoarthritis model, the hypoxic environment of the joint cavity can inhibit mTOR activity in NK cells, leading to their functional exhaustion, while pathway activators can partially restore their IFN-γ secretion capacity, reactivating the immune function of NK cells (Alter et al., 2011; Dungan et al., 2014; Rao et al., 2021). Therefore, the state of the PI3K/Akt-mTOR pathway directly determines the effector function and anti-infection vitality of NK cells and is influenced by the local microenvironment.

#### 3.2.3 Innate-like lymphocytes

Innate-like lymphocytes possess both the rapid response characteristics of innate immunity and the specificity of adaptive immunity, making them key early participants in anti-infection defense.

Natural Killer T cells (NKT cells): NKT cells recognize lipid antigens presented by CD1d molecules via their TCR and rapidly produce large amounts of cytokines in the early stages of infection, regulating the direction of the immune response. Studies indicate that the PI3K/Akt signaling pathway is crucial for the development, homeostasis, and rapid cytokine (e.g., IL-15) production of NKT cells. Akt activation effectively promotes the cytotoxic activity and immunoregulatory function of NKT cells (Watkinson et al., 2020). The PI3K/Akt pathway ensures the development and functional readiness of NKT cells as a rapid reaction force.

Mucosal-associated invariant T cells (MAIT cells): MAIT cells primarily recognize bacterial-derived vitamin B metabolites and play an important role in anti-bacterial immunity at mucosal barriers. Their activation similarly depends on TCR signaling, and co-stimulatory signals (e.g., IL-12, IL-18) can further enhance their effector functions. The PI3K/Akt-mTOR pathway, as a key signaling node downstream of the TCR, participates in regulating the activation, proliferation, and effector functions of MAIT cells, influencing their ability to produce IFN-γ, TNF-α, and granzyme B (Toubal et al., 2019). This indicates that this pathway is a core signal transduction part for MAIT cells to respond to bacterial infection and exert mucosal defense functions.

**Figure 2:**
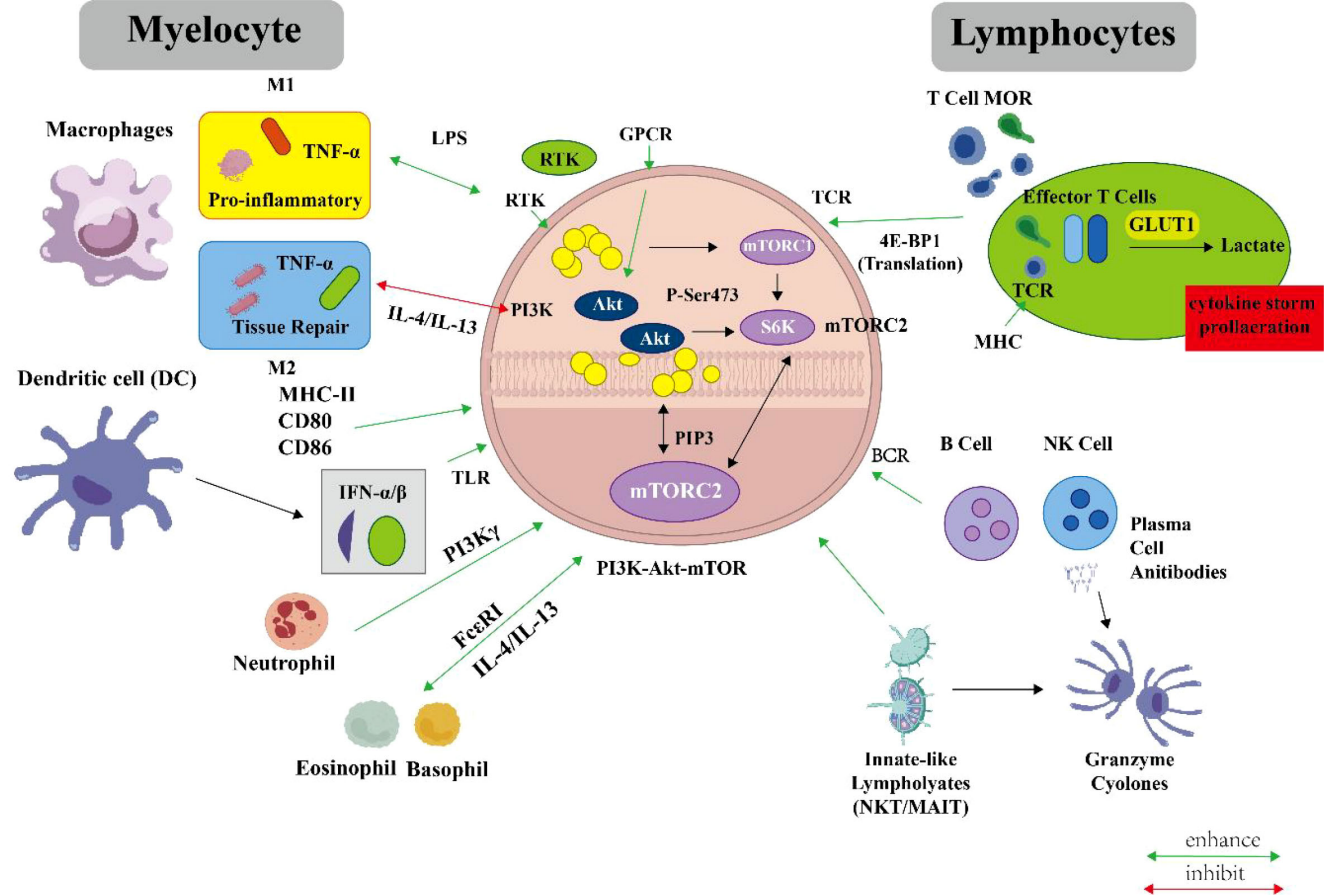
Regulatory role of the PI3K/Akt/mTOR pathway in immune cells. This schematic diagram systematically illustrates the core role of the PI3K/Akt/mTOR signaling pathway as a key hub in coordinating innate and adaptive immune responses. The pathway integrates signals from cell membrane receptors (e.g., RTK, GPCR, TLR, TCR, BCR), catalyzes the generation of PIP3, sequentially activates Akt and mTORC1/2, and subsequently regulates transcription, translation, metabolic reprogramming, and cytoskeletal reorganization, thereby determining the activation, differentiation, metabolism, and functional fate of immune cells. Left side (Myeloid cells): This pathway precisely regulates the functions of different myeloid cell subsets. In macrophages, it dynamically balances M1 (pro-inflammatory)/M2 (repair) polarization; in dendritic cells, mTORC1 drives their maturation and antigen presentation function; in plasmacytoid dendritic cells (pDCs), it promotes type I interferon (IFN-α/β) production; in neutrophils, the GPCR-PI3Kγ-PIP3 axis guides chemotaxis; in eosinophils and basophils, this pathway is involved in regulating their activation, degranulation, and roles in mediating allergic and anti-parasitic immune responses. Right side (Lymphoid cells): In adaptive immune cells, this pathway is a core driver of cell activation and differentiation. In T cells, TCR signaling strongly activates this pathway, driving metabolic reprogramming such as glycolysis, providing energy and biosynthetic materials for clonal expansion, and decisively influencing the differentiation fate of CD4^+^ T cell subsets (Th1, Th17, Treg, etc.); in B cells, BCR signaling via this pathway regulates their proliferation, germinal center reaction, and antibody secretion; the rapid effector functions of innate-like lymphocytes (e.g., NKT, MAIT cells) are also regulated by it.

γδ T cells: γδ T cells can be activated without the need for antigen presentation by classical MHC molecules and are important components of tissue-resident immune surveillance. Research shows that PI3K/Akt-mTOR signaling is involved in regulating the cytotoxic effects and cytokine production of γδ T cells, but its specific regulatory network is more complex and unique compared to αβ T cells (Silva-Santos et al., 2015). The regulation of γδ T cells suggests that the PI3K/Akt-mTOR pathway may have specialized regulatory modes in different lymphocyte subsets. (**Figure 2**).

#### 3.2.4 Summary of mechanisms and unresolved questions

In lymphocytes, the PI3K/Akt-mTOR pathway is not only a driver of activation and differentiation but also a core bridge connecting antigen signals to metabolic reprogramming. However, controversies in this field are equally significant: for example, during T cell exhaustion, is aberrant mTOR signaling a cause or a consequence of exhaustion? Existing studies are mostly based on correlations, lacking causal validation through spatiotemporally specific gene knockout in chronic infection models. Furthermore, are there inherent differences in the sensitivity of different T cell subsets (e.g., Th17 vs. Treg) to signaling via this pathway? Are these differences pre-set by epigenetic mechanisms? These questions directly relate to our ability to precisely target this pathway to modulate specific T cell functions.

## 4 Mechanisms of the PI3K/Akt-mTOR signaling pathway in infectious diseases

### 4.1 Role in bacterial infections

Pathogen manipulation of the host PI3K/Akt-mTOR pathway is not uniform. Instead, different classes of pathogens, and even different strains or life cycle stages of the same species, have evolved diverse attack strategies targeting different nodes and phases of this pathway based on their unique survival methods and pathogenic mechanisms.

#### 4.1.1 Gram-positive bacterial infections

Staphylococcus aureus, a common Gram-positive bacterium, can activate the PI3K/Akt-mTOR pathway in host endothelial and immune cells by secreting α-hemolysin (Hla), thereby exacerbating inflammatory responses and vascular damage, promoting bacterial spread (Chen et al., 2022). However, the effect of this strategy may vary depending on strain differences (e.g., Hla expression level), infection site, and host cell type, suggesting context-dependence in pathogen manipulation of the pathway. In contrast, probiotics enhance intestinal barrier function and resist pathogen invasion by inhibiting this pathway in intestinal epithelial cells, promoting secretory cell differentiation and antimicrobial peptide production (Hajialibabaei et al., 2025). These two examples together illustrate that the impact of microorganisms on the host PI3K/Akt-mTOR pathway can be either harmful or beneficial, and the ultimate outcome depends on the complex interaction of microbial species, strain characteristics, and microenvironmental context.

The virulence factor pneumolysin (PLY) of *Streptococcus pneumoniae* induces autophagy in alveolar epithelial cells by inhibiting the PI3K/Akt-mTOR pathway, enhancing the host’s ability to clear the bacteria, demonstrating a self-protective mechanism during infection (Puleston and Simon, 2014; Li et al., 2015). Studies have shown that knocking out *ply* significantly increases the intracellular survival of the bacteria, further highlighting the important role of PLY in the host-pathogen interaction (Li et al., 2015). In this case, bacterial toxin-mediated inhibition of the pathway unexpectedly activates protective host autophagy, revealing the complexity of host-pathogen interactions.

Comparing the strategies of S. aureus and S. pneumoniae reveals that the regulation of the same pathway by the same class of pathogens can produce completely opposite host outcomes (inflammatory damage vs. protective autophagy). This highlights the complexity of host-pathogen interactions: the inhibition or activation of the pathway itself is neither inherently good nor bad; its ultimate significance is determined by the spatiotemporal context and microenvironment of the infection. A key unresolved question is how pathogens evolve such precise regulatory mechanisms to “customize” pathway output to fit their survival strategy.

#### 4.1.2 Gram-negative bacterial infections

The CagA protein of *Helicobacter pylori* activates the c-Met-PI3K/Akt-mTOR axis, inhibiting macrophage autophagy (Li et al., 2017), while also promoting the secretion of pro-inflammatory factors like IL-8 (Martins et al., 2015), exacerbating gastric mucosal damage, closely linked to the development of gastritis, peptic ulcers, and even gastric cancer. In *Pseudomonas aeruginosa* infection, the bacterium enhances its adhesion and biofilm formation in airway epithelial cells by activating the PI3K/Akt pathway (Nakagawa et al., 2004; Li et al., 2015), while pathway inhibitors can significantly inhibit bacterial colonization, providing new ideas and methods for treating *P. aeruginosa* infections. In summary, various Gram-negative bacteria actively activate the host PI3K/Akt-mTOR pathway to inhibit host defense, promote their own colonization, or exacerbate tissue damage.

Compared with Gram-positive bacteria, Gram-negative bacteria (e.g., H. pylori, P. aeruginosa) tend to activate this pathway to promote their own colonization and survival, suggesting a possible association between bacterial cell wall structure and pathway manipulation strategies.

### 4.2 Role in viral infections

#### 4.2.1 DNA viruses

The HBx protein of hepatitis B virus (HBV) promotes viral replication by activating the PI3K/Akt-mTOR pathway (Yen et al., 2012; Motavaf et al., 2013; Mei et al., 2024), and actively participates in the development of hepatocellular carcinoma by upregulating factors like VEGF, severely endangering patient liver health. Kaposi’s sarcoma-associated herpesvirus (KSHV), a gamma herpesvirus, is closely associated with Kaposi’s sarcoma (KS) and B-cell lymphoproliferative disorders (Bhatt and Damania, 2012). Its encoded K1 protein can bind to the p85 subunit of PI3K, continuously activating Akt/mTOR, promoting tumor angiogenesis and viral particle release; additionally, KSHV activates the NF-κB pathway via the viral FLIP protein (Ballon et al., 2011), synergizing with PI3K/Akt-mTOR to promote B cell proliferation and survival (Hassman et al., 2011), further driving disease progression. DNA viruses employ multiple strategies to hijack and persistently activate this pathway, not only facilitating their own replication but also driving infection-related tumorigenesis.

Hepatitis B virus (HBV) HBx protein and Kaposi’s sarcoma-associated herpesvirus (KSHV) K1 protein are typical examples. They cause constitutive activation of this pathway through different mechanisms, not only promoting viral genome replication but also paving the way for virus-associated tumorigenesis by enhancing cell proliferation and survival (Tee et al., 2022; Wei et al., 2023). Notably, the K1 protein of KSHV can directly bind to the p85 regulatory subunit of PI3K to upregulate the pathway; whereas HBV HBx may function more by affecting PTEN or cooperating with Ras, suggesting that different viruses have evolved to attack different nodes of the same pathway, illustrating the diversity of hijacking mechanisms.

#### 4.2.2 RNA viruses

Rotavirus relies on the PI3K/Akt-mTOR pathway to maintain its own replication; the mTOR inhibitor rapamycin can effectively inhibit viral proliferation by inducing 4E-BP1-mediated autophagy (Thoreen et al., 2009; Coffey et al., 2016), providing a new therapeutic approach for rotavirus infection (Yin et al., 2018). Coxsackievirus B3 (CVB3) promotes autophagosome formation by activating this pathway, providing membrane structures for viral RNA replication (Xin et al., 2014), while PI3K inhibitors can significantly reduce viral titers and mitigate the damage caused by viral infection (Luo and McManus, 2012; Chang et al., 2017). Many RNA viruses cleverly exploit this pathway to create a favorable intracellular environment, such as inhibiting antiviral autophagy or hijacking autophagosomes for replication.

In COVID-19, SARS-CoV-2 exacerbates pulmonary inflammation and immune dysregulation by activating the PI3K/Akt-mTOR pathway, leading to disease progression (Basile et al., 2022). Pathway inhibitors can downregulate the overactivated inflammatory response and improve patient symptoms, offering potential therapeutic targets and strategies for COVID-19 treatment. Studies suggest that targeting the PI3K/Akt-mTOR pathway may effectively regulate the immune response in COVID-19 patients through various means, including modulating T cell function, inhibiting cytokine storms, and reducing thrombosis, thereby improving patient prognosis (Sabbah et al., 2021). This suggests that in acute severe viral infections, overactivation of this pathway is a significant driver of immunopathological damage, and its inhibitors hold multifaceted therapeutic potential.

DNA viruses (e.g., HBV, KSHV) tend to persistently activate the pathway to establish chronic infection and drive oncogenic transformation, whereas RNA viruses (e.g., rotavirus, SARS-CoV-2) more often exhibit short-term hijacking to facilitate rapid replication, reflecting fundamental differences in pathway dependence based on viral life cycles.

### 4.3 Role in parasitic infections

Protoscoleces of *Echinococcus multilocularis* activate the PI3K/Akt-mTOR pathway in macrophages, enhancing glycolysis and promoting M2 polarization, thereby creating a favorable microenvironment for parasite survival, enabling long-term parasitism in the host (Kim et al., 2017; Zhang et al., 2023). *Leishmania* parasites, on the other hand, inhibit mTOR activity in macrophages by secreting proteases (Pan et al., 2016; Wang et al., 2019), hindering antigen presentation, thus successfully evading immune clearance and surviving and multiplying within the host despite immune defenses. Parasites, by activating or inhibiting this pathway, actively shape an immunosuppressive microenvironment conducive to their own survival, which is a core strategy for establishing chronic infection.

In *Schistosoma japonicum* infection, activation of the PI3K/Akt-mTOR pathway is involved in the development of liver fibrosis. Egg antigens activate this pathway in hepatic stellate cells, promoting their proliferation and collagen synthesis, leading to liver fibrosis. Pathway inhibitors can alleviate liver tissue damage, offering new hope for the treatment of liver fibrosis associated with *S. japonicum* infection (Xiao et al., 2020). Furthermore, single-cell RNA sequencing studies have identified a pro-fibrotic *Thbs1*+ macrophage subcluster during liver fibrosis that activates hepatic stellate cells via the PI3K/Akt/mTOR signaling pathway, a mechanism validated in both mouse and human fibrotic livers, further revealing the important role of this pathway in chronic inflammation related to parasitic infections and providing crucial clues for understanding the pathogenesis of parasitic diseases and developing targeted therapeutic strategies (Cheng et al., 2023). This indicates that in chronic parasitic infections, sustained activation of the PI3K/Akt-mTOR pathway not only affects immune cells but also directly drives the pathological activation of stromal cells like fibroblasts, leading to long-term sequelae such as organ fibrosis.

Compared to bacteria and viruses, parasites are more adept at “reshaping” pathway output – by finely regulating macrophage polarization and metabolism, they shape an immunosuppressive microenvironment to achieve long-term coexistence, reflecting the unique survival strategy of multicellular pathogens.

## 5 Role in fungal infections and related research progress

In recent years, the incidence of fungal infections has been increasing annually, especially in immunocompromised populations, posing a serious threat to patient life and health. The PI3K/Akt-mTOR pathway also plays an important role during fungal infections, profoundly influencing the interaction between the host and the fungus.

Taking Aspergillus infection as an example, the hyphal and conidial forms can trigger completely opposite immune outcomes: hyphae are primarily recognized by TLR2/Dectin-1, strongly activating the PI3K/Akt/mTOR-ERK axis in host dendritic cells, driving pro-inflammatory IL-23/IL-1β production and pathogenic Th17 responses; whereas conidia activate distinct STAT3/IDO pathways via TLR3/TLR9 etc., inducing protective Th1 responses and immune regulation (Bonifazi et al., 2010). This stark contrast provides a powerful example of context-dependence in pathogen strategy – even different life stages of the same pathogen can elicit completely opposite host reactions by activating divergent downstream pathways.

*Candida albicans* can activate the PI3K/Akt pathway in platelets via integrin GP IIb/IIIa (not through TLR2/TLR4-dependent pathways), inducing P-selectin expression on the platelet surface, and promoting the release of antimicrobial peptides (e.g., PF4, CCL5) and low concentrations of TNF-α, CXCL8, directly exerting antifungal activity. Activated platelets, through GP IIb/IIIa-mediated intercellular interaction (Gautam et al., 2020), significantly enhance the phagocytic capacity of human monocytes against *C. albicans* and upregulate the expression of inflammatory cytokines, forming a “platelet-monocyte” synergistic anti-infection network (Carestia et al., 2019). The PI3K inhibitor LY294002 or the Akt inhibitor MK2206 can reverse *C. albicans*-induced platelet activation by blocking Akt Ser473 phosphorylation, reducing P-selectin expression and antimicrobial peptide release. The GP IIb/IIIa-specific antagonist tirofiban competitively binds to platelet surface GP IIb/IIIa, inhibiting platelet-*C. albicans* recognition and activation, while also blocking the enhancing effect of platelets on monocyte phagocytic function, thereby limiting excessive inflammation and fungal spread (Zheng et al., 2021). This discovery reveals the key role of the PI3K/Akt pathway in the antifungal innate immunity of non-classical immune cells (platelets) and the new role of platelets as immune regulators.

**Figure 3:**
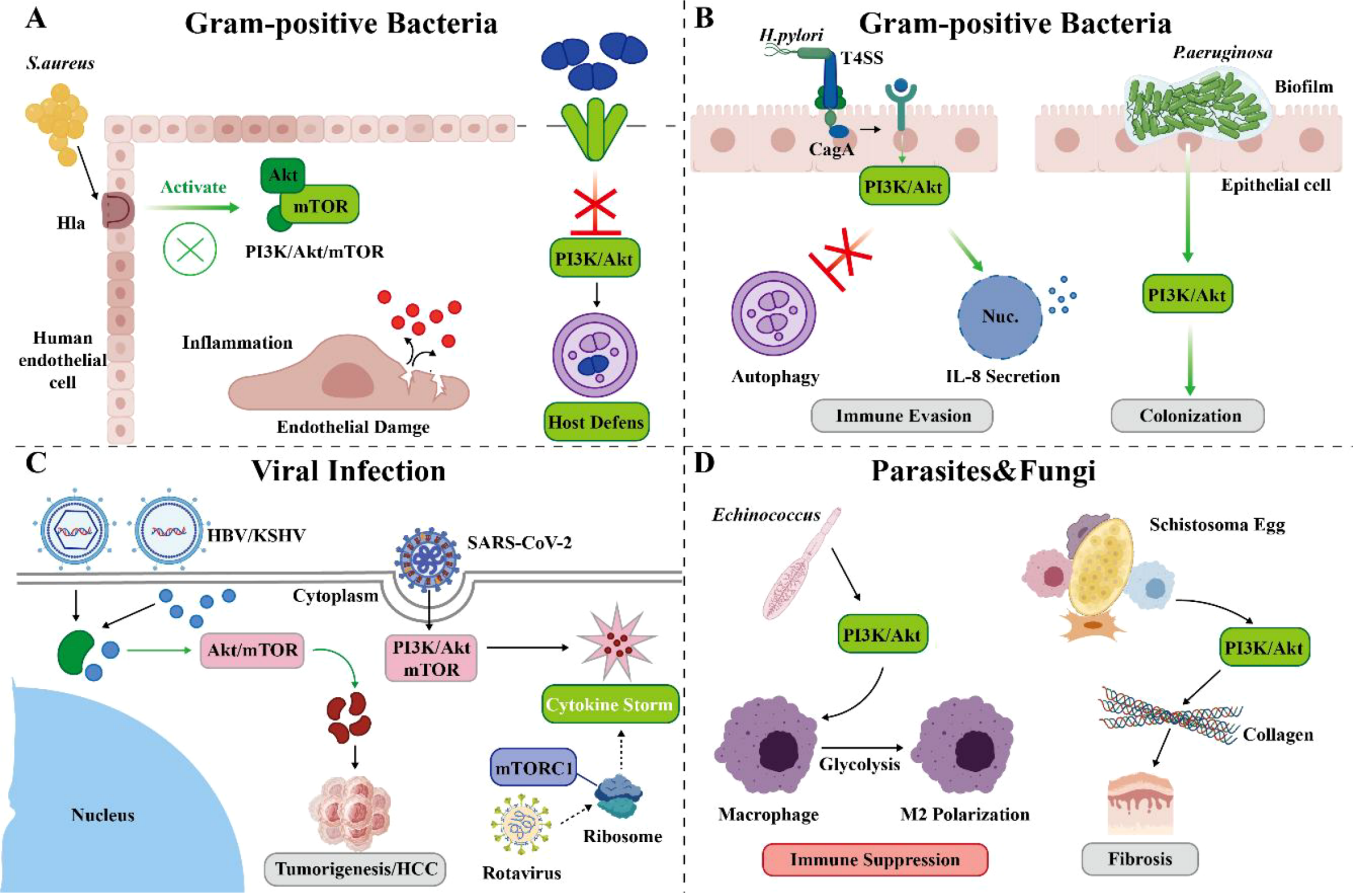
Schematic diagram of the dual role of the PI3K/Akt/mtor signaling pathway in infectious diseases. **(A)** Gram-positive bacterial infections. *Staphylococcus aureus* activates the host cell PI3K/Akt/mTOR pathway by secreting α-hemolysin (Hla), thereby promoting inflammation and causing endothelial cell damage. Conversely, *Streptococcus pneumoniae* utilizes pneumolysin (PLY) to inhibit the PI3K/Akt axis, subsequently inducing protective autophagy and enhancing host defense. **(B)** Gram-negative bacterial infections. *Helicobacter pylori* delivers the CagA protein via its type IV secretion system (T4SS), activating the c-Met-PI3K/Akt/mTOR axis, inhibiting macrophage autophagy and promoting immune evasion. *Pseudomonas aeruginosa* enhances its adhesion and biofilm formation ability, promoting colonization, by activating the PI3K/Akt pathway in epithelial cells. **(C)** Viral infections. DNA viruses such as hepatitis B virus (HBV) and Kaposi’s sarcoma-associated herpesvirus (KSHV) promote viral replication, angiogenesis, and tumorigenesis by activating the PI3K/Akt/mTOR pathway. RNA viruses like rotavirus and severe acute respiratory syndrome coronavirus 2 (SARS-CoV-2) hijack this pathway to maintain their own replication, reprogram host metabolism (e.g., glycolysis), and may lead to macrophage dysfunction and immune dysregulation. **(D)** Parasitic and fungal infections. Parasites such as *Schistosoma japonicum* eggs can promote hepatic stellate cell activation and liver fibrosis by activating the PI3K/Akt pathway. In fungal infections, pathogens can activate the PI3K/Akt/mTOR pathway in immune cells via pattern recognition receptors, thereby regulating the direction of inflammation (e.g., promoting Th2/Th17 responses) or enhancing antifungal immunity through non-classical immune cells (e.g., platelets).

In summary, the manipulation of the PI3K/Akt-mTOR pathway by pathogens exhibits remarkable diversity, adaptability, and context-dependence. The same class of pathogens (e.g., intracellular bacteria) may choose similar strategies (inhibition), but their specific molecular mechanisms and effect strengths still vary between strains. Different life stages of the same pathogen (e.g., Aspergillus hyphae vs. conidia) can even trigger completely opposite immune outcomes. Therefore, future research describing pathogen strategies should pay more attention to accurately recording variables such as strain background, infection phase, cell type, and microenvironment, avoiding overgeneralization of observations under specific conditions as ‘universal laws’. (**Figure 3**).

### 5.1 Summary of mechanisms and comparative analysis

Placing the strategies of different pathogens within the same framework reveals profound evolutionary logic: viruses tend to “hijack” (activate the pathway to utilize host resources), intracellular bacteria tend to “paralyze” (inhibit the pathway to evade clearance), and multicellular pathogens tend to “reshape” (finely regulate the pathway to shape a tolerogenic microenvironment). However, this framework still faces many challenges. A prominent contradiction is: why do intracellular bacteria like Listeria and Mycobacterium tuberculosis differ in their manipulation strategies? This may relate to their specific intracellular niches (cytosol vs. phagosome), but direct evidence is lacking. Another contentious point is: is pathogen manipulation of the pathway an active, finely designed strategy, or a byproduct of host cellular stress responses? This touches upon the fundamental difficulty of defining causality. Future research needs to combine evolutionary biology and systems biology approaches to delve into the evolutionary drivers behind these strategies from the perspective of “convergent evolution” and “divergent selection” in pathogen genomes.

## 6 Therapeutic prospects and translational challenges of targeting the PI3K/Akt-mTOR pathway

The dual nature of the pathway is undoubtedly a major challenge in clinical application. For example, in the treatment of sepsis, inhibiting the PI3K/Akt-mTOR pathway may reduce inflammatory damage, but it could simultaneously weaken the host’s ability to clear pathogens (Xiahou et al., 2017; Li et al., 2023), increasing the risk of infection spread. *Preclinical studies indicate that mTOR inhibitors (e.g., rapamycin) can inhibit the replication of various viruses, including rotavirus and cytomegalovirus (Yin et al., 2018; Zhou et al., 2020), through mechanisms involving the induction of autophagy and regulation of host cell metabolism. In the treatment of COVID-19, retrospective studies suggest that mTOR inhibitors may alleviate disease severity by suppressing excessive inflammatory responses (Chapman et al., 2015). In sepsis models, PI3K/mTOR inhibitors can reduce excessive inflammation and multi-organ damage (Sha et al., 2021), but inhibiting this pathway in the early stages of infection might impair neutrophil function and increase infection risk (Hirsch et al., 2000).

Applying this strategy clinically faces multiple challenges. This pathway simultaneously regulates host defense and immunopathology; inhibition, while potentially reducing inflammation, may compromise pathogen clearance (Li et al., 2015). Experience from oncology provides important warnings: mTOR inhibitors (everolimus, sirolimus) increase the risk of opportunistic infections in organ transplant patients (Narasimhan et al., 2016); patients treated with the PI3Kδ inhibitor idelalisib for leukemia show significantly higher incidence rates of CMV and Pneumocystis jirovecii pneumonia (Zhou et al., 2018). Pathogen resistance mechanisms are equally concerning – *S. aureus* can antagonize PI3K inhibitors by upregulating PTEN expression (Buchkovich et al., 2008), and viruses may develop resistance through mutations in effector proteins or activation of compensatory pathways (Bhatt and Damania, 2012). Furthermore, first-generation pan-PI3K/mTOR inhibitors are often associated with side effects such as hyperglycemia and dyslipidemia, with narrow therapeutic windows (Zhang et al., 2019).

Addressing these challenges requires a shift from “broad-spectrum inhibition” to “precision intervention”: isoform-specific targeting (PI3Kδ inhibitors targeting lymphocytes, PI3Kγ inhibitors targeting myeloid cells (Zhou et al., 2018), timing-dependent intervention (avoid inhibition early in infection to ensure pathogen clearance, intervene in mid-to-late stages to control immunopathology), branch-selective regulation (selectively targeting mTORC1 while preserving mTORC2-mediated cell survival signals (Chen et al., 2022), and combination therapies (with antimicrobial agents or immune checkpoint inhibitors). Translating mechanistic insights into cell-specific and timing-dependent innovative therapies is the essential path for this field towards clinical application.

## 7 Conclusion and outlook

The PI3K/Akt-mTOR signaling pathway, as a core intracellular regulatory network, plays a decisive role in the progression and outcome of infectious diseases. The host enhances immune defense by activating this pathway, while pathogens achieve immune evasion by targeting key molecules within the pathway. Furthermore, in-depth study of the mechanisms of this pathway in different pathogen infections provides key targets for developing novel anti-infection strategies. Currently, targeted therapy research based on the PI3K/Akt-mTOR signaling pathway has garnered significant attention. Two main intervention approaches are employed: the first involves designing inhibitors targeting key components of this pathway, such as PI3K, Akt, and mTOR; the second focuses on developing isoform-specific inhibitors or pathway modulators. Encouragingly, the PI3K/Akt-mTOR signaling pathway has been demonstrated as a potential target for immunotherapy in various infectious diseases.

In conclusion, the PI3K/Akt-mTOR signaling pathway is widely regarded as a potential target for immunotherapy to control infections. Therefore, utilizing cutting-edge technologies to deeply analyze its regulatory heterogeneity at the single-cell level, developing highly selective modulators, exploring cross-talk with other pathways, and strengthening research on pathogen evasion mechanisms are of great significance. Through multidisciplinary collaboration, targeting the PI3K/Akt-mTOR pathway holds promise as an important breakthrough in combating infectious diseases.
